# The Application of Fulvic Acid Can Enhance the Performance of Rice Seedlings Under Low-Nitrogen Stress

**DOI:** 10.3390/plants14182892

**Published:** 2025-09-18

**Authors:** Ke Ma, Yuanyuan Zhou, Zexin Qi

**Affiliations:** 1 Agronomy College, Jilin Agricultural Science and Technology College, Jilin 132101, China; 2 Agronomy College, Jilin Agricultural University, Changchun 130118, China

**Keywords:** fulvic acid, rice seedlings, low nitrogen, photosynthesis, nitrogen metabolism, antioxidant

## Abstract

Fulvic acid’s potential to enhance plant growth has been recognized, but its effects on plant growth and nutrient uptake under nutrient stress remain unclear. This experiment investigated the effects of fulvic acid at concentrations of 0 mg L^−1^ (T1), 30 mg L^−1^ (T2), 60 mg L^−1^ (T3), 90 mg L^−1^ (T4), 120 mg L^−1^ (T5), and 150 mg L^−1^ (T6) on the growth performance of two rice varieties—Jikedao 654 (J 654) and Jiyang 100 (J 100)—under low-nitrogen stress in a hydroponic system. The effects of different fulvic acid application rates on the growth and photosynthetic characteristics, the key enzymes of nitrogen metabolism, antioxidant properties, and the osmotic adjustment substances of rice under low-nitrogen stress were evaluated. The results indicated that the addition of an appropriate concentration of fulvic acid could enhance the growth performance of J 654 and J 100 under low-nitrogen stress. Compared to T1 treatment, the total dry weight and nitrogen accumulation of rice showed greater increases in response to T3 and T4 treatments. The photosynthetic pigment content increased, photosynthesis was enhanced, and the net photosynthetic rate (Pn), stomatal conductance (Gs), intercellular CO_2_ concentration (Ci), and transpiration rate (Tr) were improved. The activities of key enzymes in nitrogen metabolism, including nitrate reductase (NR), glutamine synthetase (GS), glutamate oxaloacetate transaminase (GOT), and glutamate pyruvate transaminase activity (GPT), were enhanced, thereby improving the capacity for nitrogen uptake and assimilation. The addition of fulvic acid also enhanced the antioxidant capacity, increased the superoxide dismutase (SOD), peroxide (POD) and catalase (CAT) activity and decreased the toxic effects of ROS, the production rate of O_2_^−^, and the hydrogen peroxide (H_2_O_2_) and malondialdehyde (MDA) content. The low-nitrogen stress was alleviated, thereby reducing the proline and soluble sugars content. Overall, it was demonstrated that adding an appropriate concentration (60–90 mg L^−1^) of fulvic acid under low-nitrogen stress has a positive impact on the growth and development of rice. Our findings provide a theoretical basis for the application of fulvic acid in alleviating low-nitrogen stress in rice.

## 1. Introduction

Rice (*Oryza sativa* L.) is a staple food crop for more than half of the global population [1,2]. With the continuous growth of the population, global rice production needs to increase to 116 million tons by 2035 to meet the rising demand [3]. Achieving this is crucial for global food security, especially in regions where rice is both a staple food and a source of income [4]. However, the intensification of rice production still faces significant challenges. While enhancing the yield and quality of rice, it is also essential to ensure the sustainable development of the environment [5,6]. Various methods such as high-yield varieties, improved planting density, and optimized irrigation strategies have significantly enhanced productivity. Conventional fertilizers, particularly nitrogen fertilizers, are indispensable for increasing crop yield and quality; however, there are considerable concerns surrounding their use [7]. When used in paddy fields, conventional fertilizers may lead to significant nutrient loss, resulting in soil degradation, water pollution, and ammonia volatilization [8,9]. Over the past few decades, the global nitrogen fertilizer input has increased by 800% [10], successfully enhancing rice yield. However, further application of nitrogen fertilizer does not necessarily effectively improve rice yield and nitrogen-use efficiency (NUE) [11,12]. The nitrogen-use efficiency of rice may reach 20–30%, while 70–80% of the applied nitrogen fertilizer remains unutilized [13], leading to a low nitrogen-use efficiency [14]. Therefore, reducing the nitrogen fertilizer application while maintaining yield is crucial for the sustainable production of rice.

Humus, comprising humic acid, humin, and fulvic acid, is an organic mixture that accumulates in soil through a series of chemical reactions involving microbial decomposition, transformation, and the participation of plant and animal residues. Numerous studies have demonstrated that humus plays an effective role in stimulating plant growth, enhancing nutrient absorption, and improving crop yield and quality [15,16,17]. Fulvic acid is a highly bioactive component of humus which is characterized by its low molecular weight, good water solubility, and ease of absorption by plants [18,19]. It is primarily composed of soluble organic compounds and serves as a bioactivator [20]. In addition, fulvic acid can influence various physiological processes, such as plant growth, nutrient uptake, protein and nucleic acid synthesis, photosynthesis, and respiration [21,22]. For example, it mitigates lead toxicity in plants by reducing lead absorption and maintaining the normal growth and development of the plants [23].

Relevant studies have demonstrated the efficacy of fulvic acid in agriculture. Fulvic acid regulates the ascorbic acid metabolism, glutathione metabolism, and flavonoid biosynthesis in tea plants, thereby enhancing their antioxidant defense capacity under drought stress [24]. The application of fulvic acid can alleviate the growth inhibition of continuously cropping potato seedlings, thereby functioning similarly to hormones and environmental response regulators, promoting plant growth and development [25]. Studies have shown that fulvic acid can enhance the growth, yield, and NUE of maize through its synergistic effects in improving nitrogen uptake, promoting photosynthesis, enhancing carbon/nitrogen metabolic processes, and increasing enzyme activity [26]. In a pot experiment with maize, foliar application of fatty acids was found to increase leaf area, augment the number of leaves, and extend the length of maize ears, thereby enhancing yield and biomass [27]. Lv et al. [28] found that the addition of an appropriate concentration of fulvic acid could promote phosphorus uptake in rice, alleviate low-phosphorus stress, and improve the growth conditions of rice under low-phosphorus stress. In summary, research on the role fulvic acid plays in promoting plant growth and nutrient absorption and the effects of fulvic acid under nutrient stress conditions is limited.

Although previous studies have demonstrated the potential of fulvic acid in enhancing crop stress resistance, most research has focused on drought or saline-alkali stress, with limited attention given to low-nitrogen conditions. The aim of this study was to screen the effect of different concentrations of fulvic acid on rice seedlings under low-nitrogen stress conditions, with the purpose to prospect stress mitigation. To this end, two objectives were established. The first was to assess the influence of fulvic acid application on agronomic traits, photosynthetic characteristics, the activities of nitrogen metabolism-related enzymes, and antioxidant properties. The second was to define the physiological mechanisms of fulvic acid that promotes rice growth in low-nitrogen stress conditions.

## 2. Results

### 2.1. The Effect of Fulvic Acid on Rice Growth Under Low-Nitrogen Stress

**Figure 1:**
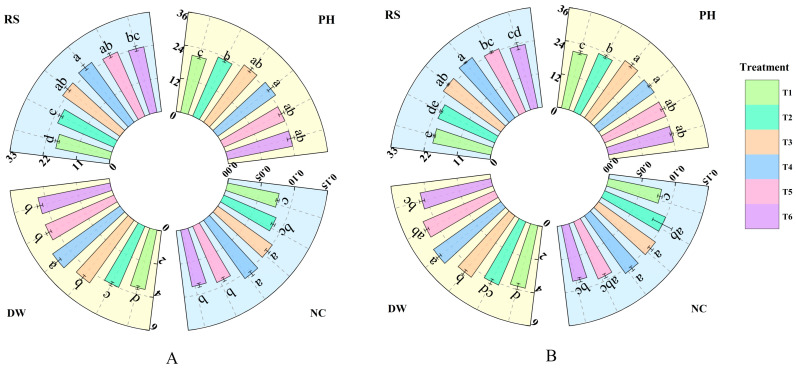
The effects of different concentrations of fulvic acid on the growth of (**A**) (J 654) and (**B**) (J 100) under low-nitrogen stress. PH: plant height (cm); RS: number of roots; DW: total dry weight (g/plant); and NC: nitrogen accumulation (g/plant). Values are presented as means ± SD, *n* = 9. Different letters on the column represent significant differences (*p* < 0.05) between different treatments of the same rice varieties, based on the least significant difference (LSD) test.

Treatment with different concentrations of fulvic acid affected the growth of rice (Figure 1). As the fulvic acid concentration increased, the plant height, root number, total dry weight, and nitrogen accumulation of J 654 and J 100 showed an increasing trend. The plant height of J 654 and J 100 under T2, T3, T4, T5, and T6 treatments was significantly (*p* < 0.05) higher than that under T1 treatment. J 654 increased by 10.66%, 20.93%, 22.46%, 14.39%, and 14.25%, respectively, while J 100 increased by 9.45%, 21.51%, 18.34%, 15.85%, and 14.87%, respectively. Among them, there was no significant difference in the root numbers between treatments T3, T4, T5, and T6 for J 654 and J 100. The root numbers of J 654 and J 100 under T3, T4, T5, and T6 treatments were significantly (*p* < 0.05) higher than that under T1 treatment. J 654 increased by 26.85%, 32.14%, 25.68%, and 20.25%, respectively, while J 100 increased by 18.61%, 24.43%, 15.19%, and 8.99%, respectively. The total dry weights of J 654 and J 100 under T3, T4, T5, and T6 treatments were significantly (*p* < 0.05) higher than that under T1 treatment. J 654 increased by 26.98%, 36.56%, 23.53%, and 21.47%, respectively, while J 100 increased by 18.43%, 29.69%, 22.62%, and 13.45%, respectively. The nitrogen accumulation of J 654 and J 100 under T3, T4, and T5 treatments was significantly (*p* < 0.05) higher than that under T1 treatment. J 654 increased by 27.30%, 35.90%, and 16.15%, respectively, while J 100 increased by 28.82%, 30.83%, and 14.70%, respectively. J 654 and J 100 exhibited the highest nitrogen accumulation under the T3 and T4 treatments.

### 2.2. The Effect of Fulvic Acid on Chlorophyll Content in Rice Leaves Under Low-Nitrogen Stress

**Figure 2:**
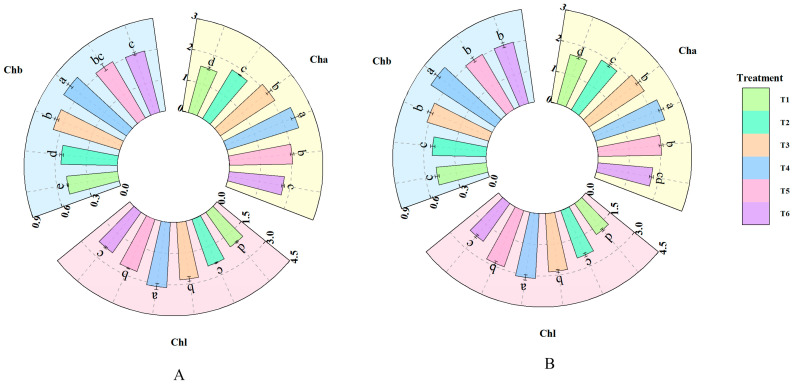
The effects of different concentrations of fulvic acid on the chlorophyll content of (**A**) (J 654) and (**B**) (J 100) under low-nitrogen stress. Cha: chlorophyll a content (mg/g); Chb: chlorophyll b content (mg/g); and Chl: total chlorophyll content (mg/g). Values are presented as means ± SD, *n* = 9. Different letters on the column represent significant differences (*p* < 0.05) between different treatments of the same rice varieties, based on LSD test.

As shown in Figure 2, the treatment with different concentrations of fulvic acid affected the chlorophyll content in rice leaves. The contents of Cha, Chb, and Chl in both J 654 and J 100 increased with the increasing concentration of fulvic acid. The Cha content of J 654 and J 100 under T2, T3, T4, and T5 treatments was significantly (*p* < 0.05) higher than that under T1 treatment. J 654 increased by 18.41%, 35.35%, 56.06%, and 31.40%, respectively, while J 100 increased by 14.55%, 29.64%, 42.08%, and 24.82%, respectively. The Chb content of J 654 and J 100 under T3, T4, and T5 treatments was significantly (*p* < 0.05) higher than that under T1 treatment. J 654 increased by 36.30%, 46.69%, and 27.34%, respectively, while J 100 increased by 53.72%, 70.85%, and 56.61%, respectively. The Chl content of J 654 and J 100 under T3, T4, and T5 treatments was significantly (*p* < 0.05) higher than that under T1 treatment. J 654 increased by 35.58%, 53.79%, and 30.42%, respectively, while J 100 increased by 27.95%, 52.19%, and 25.48%, respectively. Among them, J 654 and J 100 exhibited the highest Cha, Chb, and Chl contents under the T4 treatments.

### 2.3. The Effect of Fulvic Acid on Photosynthetic Parameters of Rice Leaves Under Low-Nitrogen Stress

**Figure 3:**
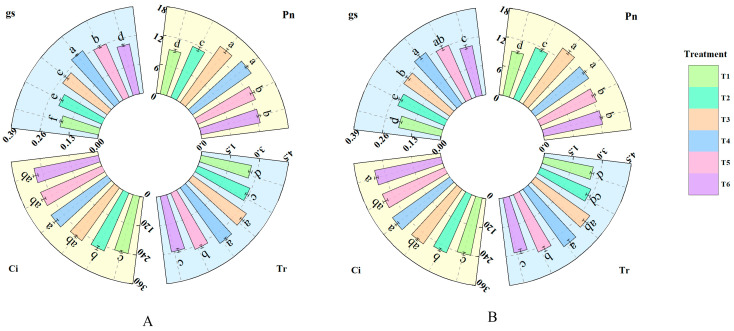
The effects of different concentrations of fulvic acid on the photosynthetic parameters of (**A**) (J 654) and (**B**) (J 100) under low-nitrogen stress. Pn: net photosynthetic rate (μmol·m^−2^s^−1^); Gs: stomatal conductance (mol·m^−2^s^−1^); Ci: intercellular CO_2_ concentration (μmol·mol^−1^); and Tr: transpiration rate (mmol·m^−2^s^−1^). Values are presented as means ± SD, *n* = 9. Different letters on the column represent significant differences (*p* < 0.05) between different treatments of the same rice varieties, based on LSD test.

As shown in Figure 3, treatments with different concentrations of fulvic acid affected the photosynthesis of rice leaves. Pn, Gs, Ci, and Tr all increased with the increasing concentration of fulvic acid. The Pn of J 654 and J 100 under T2, T3, T4, T5, and T6 treatments was significantly (*p* < 0.05) higher than that under T1 treatment. J 654 increased by 22.32%, 52.47%, 59.05%, 38.66%, and 33.15%, respectively, while J 100 increased by 23.49%, 52.17%, 45.24%, 35.14%, and 32.19%, respectively. The Pn under treatments T3 and T4 was significantly higher than that under other treatments. The Gs of J 654 and J 100 under T2, T3, T4, T5, and T6 treatments was significantly (*p* < 0.05) higher than that under T1 treatment. J 654 increased by 19.71%, 39.53%, 62.03%, 47.84%, and 29.74%, respectively, while J 100 increased by 17.18%, 30.24%, 45.19%, 34.36%, and 16.84%, respectively. The Gs under treatment T4 was significantly higher than that under other treatments. The Ci of J 654 and J 100 under T2, T3, T4, T5, and T6 treatments was significantly (*p* < 0.05) higher than that under T1 treatment. J 654 increased by 8.32%, 11.80%, 17.93%, 14.66%, and 12.81%, respectively, while J 100 increased by 8.43%, 14.66%, 20.13%, 15.41%, and 15.95%, respectively. However, there was no significant difference in Ci among T3, T4, T5, and T6 treatments. The Tr of J 654 and J 100 under T3, T4, and T5 treatments was significantly (*p* < 0.05) higher than that under T1 treatment. J 654 increased by 28.56%, 33.10%, and 22.15%, respectively, while J 100 increased by 32.46%, 39.93%, and 23.97%, respectively.

### 2.4. The Effect of Fulvic Acid on Nitrogen Metabolism Enzyme Activities in Rice Leaves Under Low-Nitrogen Stress

**Figure 4:**
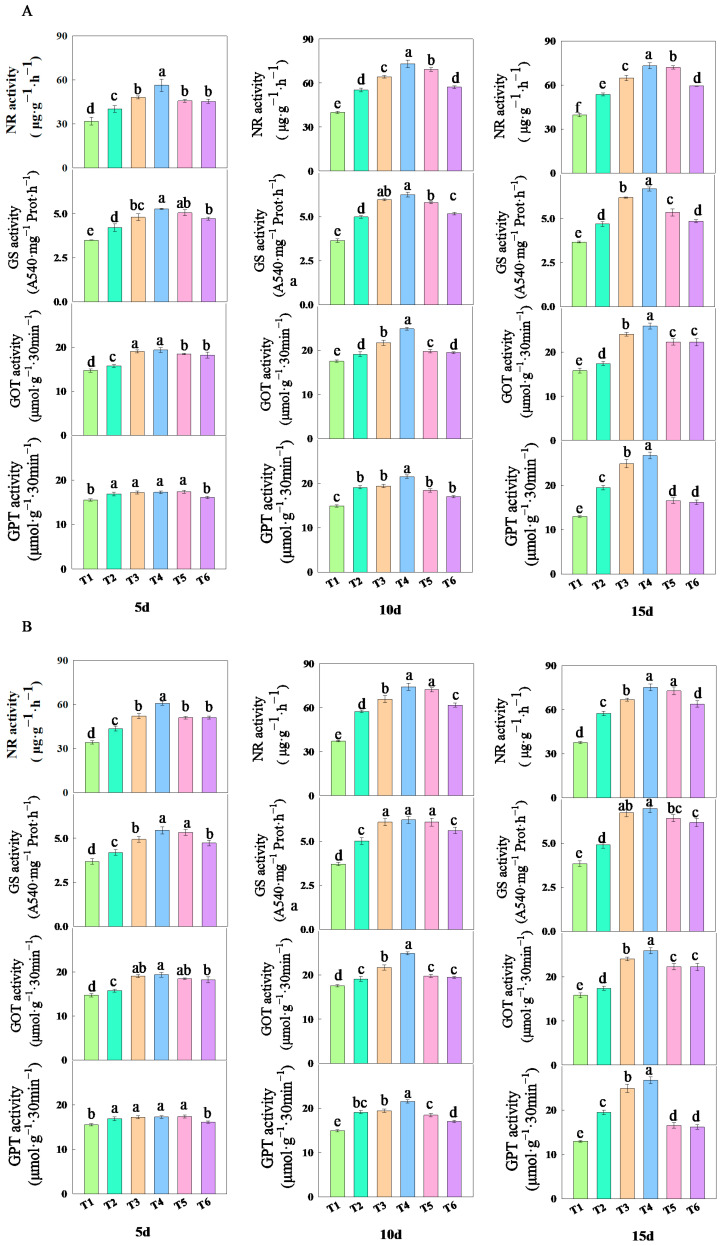
The effects of different concentrations of fulvic acid on the nitrogen metabolism enzymes of (**A**) (J 654) and (**B**) (J 100) under low-nitrogen stress. Values are presented as means ± SD, *n* = 9. Different letters on the column represent significant differences (*p* < 0.05) between different treatments of the same rice varieties, based on LSD test.

As shown in Figure 4, the treatment with different concentrations of fulvic acid affected the changes in nitrogen metabolism enzyme activities in rice leaves. The activities of NR, GS, GOT, and GPT in the leaves increased with the increase in the fulvic acid concentration. The changes in the NR, GS, GOT, and GPT activity vary under different treatments (Table S1). Among them, the activity of NR, GS, GOT, and GPT all reached their highest levels after 15 days of treatment, while the concentrations of T3 and T4 remained at their peak values. After 15 days of treatment, the NR activity of J 654 and J 100 under T2, T3, T4, T5, and T6 treatments was significantly (*p* < 0.05) higher than that under T1 treatment. J 654 increased by 35.73%, 64.16%, 85.27%, 82.49%, and 50.25%, respectively, while J 100 increased by 53.27%, 78.28%, 100.43%, 94.26%, and 70.18%, respectively; the GS activity of J 654 was significantly (*p* < 0.05) increased by 27.90%, 68.68%, 81.95%, 45.55%, and 32.03%, respectively, while J 100 showed a significant (*p* < 0.05) increase by 27.60%, 74.92%, 81.08%, 67.07%, and 60.70%; the GOT activity of J 654 was significantly (*p* < 0.05) increased by 14.17%, 44.12%, 56.75%, 29.26%, and 29.98%, respectively, while J 100 showed a significant (*p* < 0.05) increase by 9.18%, 51.95%, 63.62%, 40.82%, and 40.58%; and the GPT activity of J 654 was significantly (*p* < 0.05) increased by 45.22%, 62.03%, 79.37%, 27.25%, and 26.11%, respectively, while J 100 showed a significant (*p* < 0.05) increase by 50.05%, 91.83%, 106.36%, 27.52%, and 24.74%.

### 2.5. The Effect of Fulvic Acid on the Antioxidant Characteristics of Rice Leaves Under Low-Nitrogen Stress

**Figure 5:**
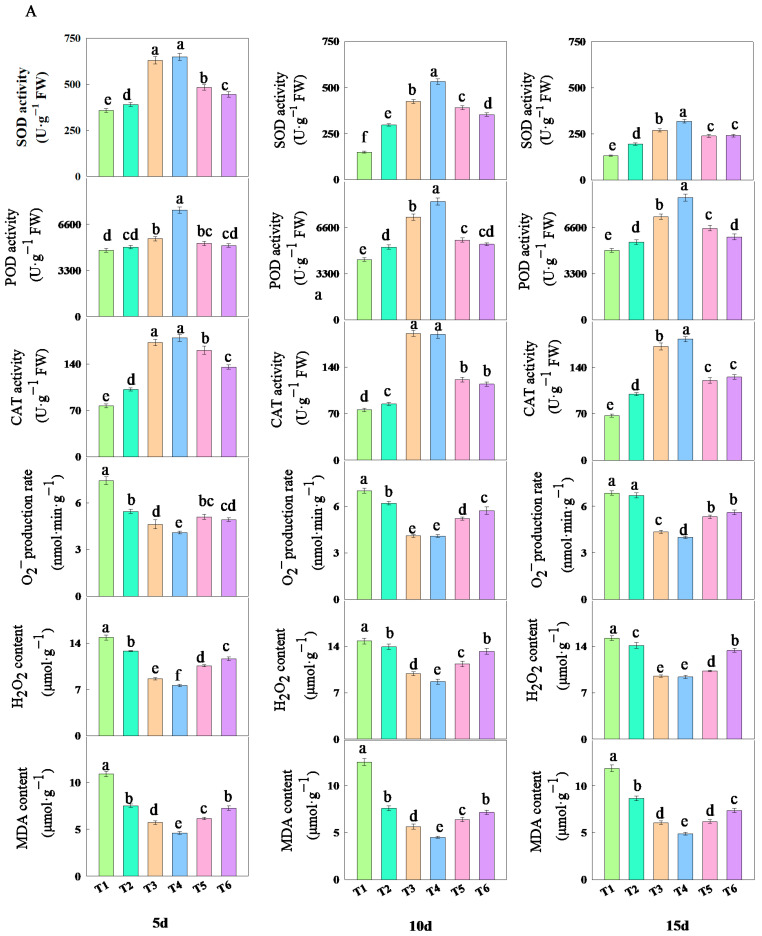

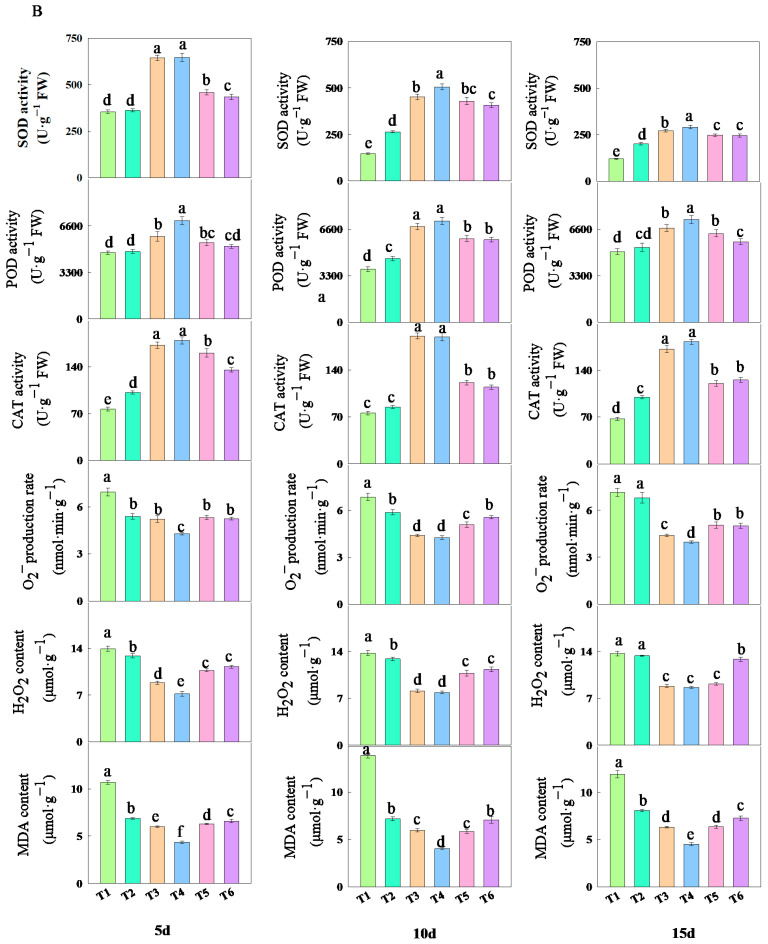
The effects of different concentrations of fulvic acid on the antioxidant properties of (**A**) (J 654) and (**B**) (J 100) under low-nitrogen stress. Values are presented as means ± SD, *n* = 9. Different letters on the column represent significant differences (*p* < 0.05) between different treatments of the same rice varieties, based on LSD test.

The treatment with different concentrations of fulvic acid affected the antioxidant properties of rice leaves (Figure 5). As the concentration of fulvic acid increased, the activities of SOD, POD, and CAT in the leaves increased. Among them, the activities of SOD and CAT decreased with the number of treatment days, while the activity of POD slightly increased with the number of treatment days. The activities of SOD, POD, and CAT in J 654 and J 100 were consistently the highest under T3 and T4 treatments and always the lowest under T1 treatment (Table S2). The SOD activity registered higher values in the first 5 days and showed a decrease by the last 15-day interval. However, the SOD, POD, and CAT activities under T3 and T4 treatments consistently maintained the highest values. After 15 days of treatment, compared with T1 treatment, the increase in the SOD activity of J 654 under other treatments ranged from 47.57 to 141.40%; the increase for J 100 ranged from 68.44 to 142.94%; the increase in the POD activity of J 654 under other treatments ranged from 12.01 to 75.99%; the increase for J 100 ranged from 6.10 to 45.62%; the increase in the CAT activity of J 654 under other treatments ranged from 48.60 to 172.55%; and the increase for J 100 ranged from 37.96 to 160.72%.

With the increase in the fulvic acid concentration, the O_2_^−^ production rate, H_2_O_2_ content, and MDA content in the leaves decreased (Figure 5). Among them, the O_2_^−^ production rate, H_2_O_2_ content, and MDA content showed a slight increase with the number of treatment days. Under T1 treatment, J 654 and J 100 consistently exhibited the highest rates of the O_2_^−^ production rate and H_2_O_2_ and MDA contents. Conversely, under T4 treatment, they consistently showed the lowest rates of O_2_^−^ production and H_2_O_2_ and MDA content (Table S2). Among them, the O_2_^−^ production rate, H_2_O_2_ content, and MDA content under T3 and T4 treatments consistently remained at the lowest levels. After five days of treatment, compared with T1 treatment, the decrease in O_2_^−^ production rates of J 654 under other treatments ranged from 26.47 to 44.90%; the decrease for J 100 ranged from 22.37 to 8.24%; the decrease in the H_2_O_2_ content of J 654 under other treatments decreased from 13.77 to 48.74%; the decrease for J 100 ranged from 7.77 to 48.54%; the decrease in the MDA content of J 654 under other treatments ranged from 31.00 to 57.4%; and the decrease for J 100 ranged from 35.83 to 59.36%. After 15 days of treatment, compared with the T1 treatment, the decrease in O_2_^−^ production rates of J 654 under other treatments ranged from 2.24 to 41.51%; the decrease for J 100 ranged from 4.87 to 44.29%; the decrease in the H_2_O_2_ content of J 654 under other treatments ranged from 7.26 to 38.40%; the decrease for J 100 ranged from 2.37 to 36.87%; the decrease in the MDA content of J 654 under other treatments ranged from 26.97 to 58.74%; and the decrease for J 100 ranged from 32.15 to 62.11%.

### 2.6. The Effect of Fulvic Acid on Osmotic Adjustment Substances in Rice Leaves Under Low-Nitrogen Stress

**Figure 6:**
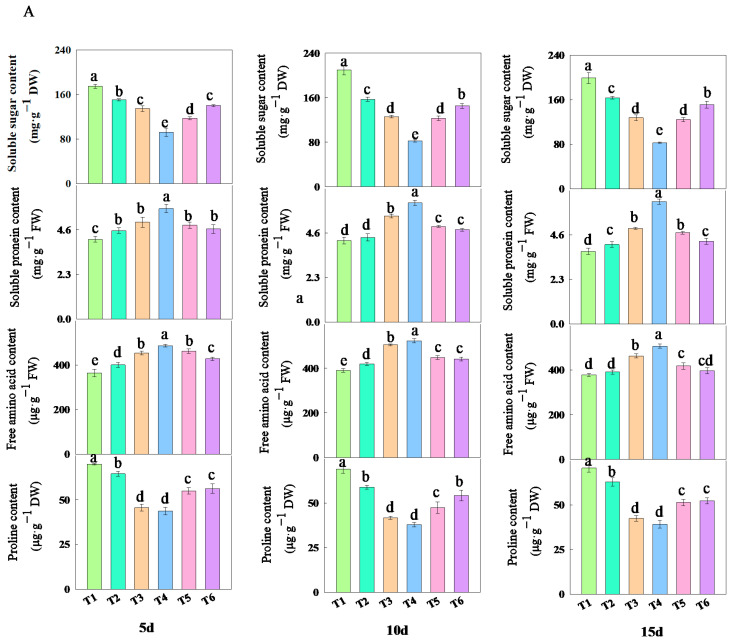

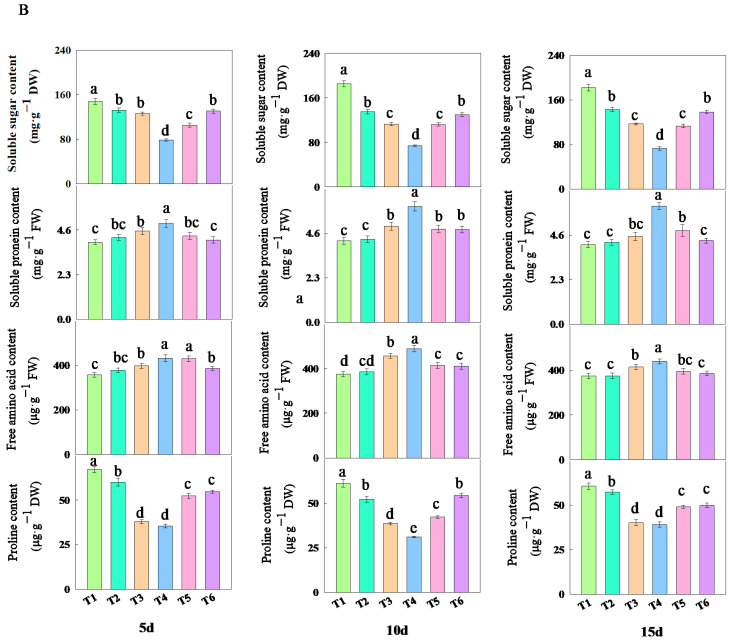
The effects of different concentrations of fulvic acid on the osmotic adjustment substances of (**A**) (J 654) and (**B**) (J 100) under low-nitrogen stress. Values are presented as means ± SD, *n* = 9. Different letters on the column represent significant differences (*p* < 0.05) between different treatments of the same rice varieties, based on LSD test.

As shown in Figure 6, treatments with different concentrations of fulvic acid affected the changes in the content of osmotic adjustment substances in rice leaves. The addition of fulvic acid decreased the content of soluble sugars and proline while increasing the content of soluble proteins and free amino acids. Among them, the content of soluble sugars and proline under T3 and T4 treatments consistently remained at the lowest levels, while the content of soluble proteins and free amino acids consistently maintained the highest levels (Table S3). After 15 days of treatment, compared with the T1 treatment, the decrease in soluble sugar content of J 654 under other treatments ranged from 17.84 to 58.34%; the decrease for J 100 ranged from 21.33 to 59.93%; the decrease in the proline content of J 654 under other treatments ranged from 11.01 to 44.42%; the decrease for J 100 ranged from 5.55 to 35.47%; the increase in the soluble protein content of J 654 under other treatments ranged from 9.40 to 68.31%; the increase for J 100 ranged from 3.02 to 48.56%; the increase in the free amino acid content of J 654 under other treatments ranged from 3.38 to 33.96%; and the increase for J 100 ranged from 0.14 to 17.42%.

### 2.7. Principal Component Analysis

**Figure 7:**
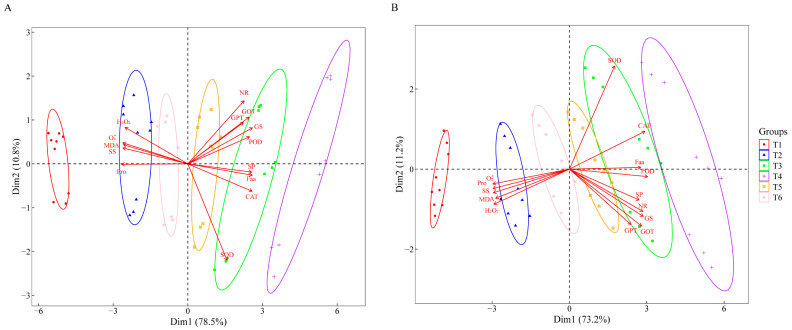
Principal component analysis of nitrogen metabolism and antioxidant indices in (**A**) (J 654) and (**B**) (J 100). Note: SS: soluble sugar; SP: soluble protein; Faa: free amino acid; and Pro: proline.

Figure 7 shows the results of the principal component analysis of the nitrogen metabolism and antioxidant indicators. The Dim 1 of J 654 was 78.5%, and the Dim 2 was 10.8%. The Dim 1 of J 100 was 73.2%, and the Dim 2 was 11.2%. J 654 and J 100 showed significant negative correlations between O_2_^−^, H_2_O_2_, MDA, SS, and Pro with NR, GS, GOT, GPT, POD, CAT, SP, and Faa.

### 2.8. Correlation Analysis

**Figure 8:**
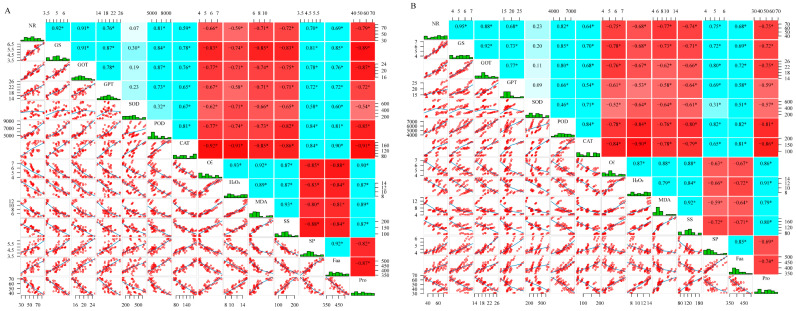
Correlation analysis of nitrogen metabolism and antioxidant indicators between (**A**) (J 654) and (**B**) (J 100). *—significant difference at 0.05 level (*p* < 0.05). The red background of the data in the figure shows a negative correlation, while the blue background shows a positive correlation.

Figure 8 showed the correlations between the rice nitrogen metabolism and antioxidant indicators. The NR, GS, GOT, and GPT of J 654 and J 100 showed a significant positive correlation with POD, CAT, SP, and Faa and a significant negative correlation with O_2_^−^, H_2_O_2_, MDA, SS, and Pro.

## 3. Discussion

Nitrogen insufficiency has many negative effects on plants. Generally, it decreases plant growth by affecting physiological processes, such as decreasing the presence of photosynthetic pigments in leaves and the activity of hormones and enzymes. This, in turn, impacts photosynthesis and, ultimately, crop production [29,30]. Growth inhibition may be caused by certain factors, such as osmotic pressure, ion toxicity, nutrient uptake limitations, decreased photosynthesis, and ion accumulation in plant tissues. Exogenous hormones play a significant role in the response to various abiotic stresses. Relevant studies have shown that fulvic acid has a positive impact on promoting crop development and growth. Fulvic acid can enhance plant growth, nutrient uptake, photosynthetic efficiency, and crop resistance [31,32]. In this study, the addition of fulvic acid increased the plant height, root number, total dry weight, and nitrogen accumulation of rice. An appropriate concentration of fulvic acid can alleviate the adverse effects of low-nitrogen stress on rice growth and development. The plant height, root number, total dry weight, and nitrogen accumulation decreased under the T5 and T6 treatments compared to those under the T4 treatment. The application of the optimal concentration of fulvic acid (90 mg L^−1^) has a positive impact on plant growth, which was conducive to mitigating the inhibition of plant growth caused by low nitrogen.

During the process of plant growth, photosynthesis is relatively sensitive to environmental changes. A significant portion of assimilated nitrogen in plants is also allocated to photosynthesis. Leaf chloroplasts are the primary sites of photosynthesis. Chlorophyll a and chlorophyll b, as the main light-absorbing and transmitting pigments (accessory pigments), play crucial roles in enhancing light capture efficiency. A reduction in the nitrogen supply affects the normal growth of rice seedlings and disrupts the balance of pigment metabolism and influences of chlorophyll a and chlorophyll b content [33]. In this study, the increase in fulvic acid concentration enhanced the contents of chlorophyll a, chlorophyll b, and total chlorophyll. However, excessively high concentrations of fulvic acid were detrimental to the increase in chlorophyll a, chlorophyll b, and total chlorophyll content. The chlorophyll a, chlorophyll b, and total chlorophyll content consistently remained the highest under the T4 treatment. This indicates that an appropriate concentration of fulvic acid is beneficial for the synthesis of photosynthetic pigments in leaves, enhancing the capacity for light energy capture. The nitrogen used in photosynthesis is primarily allocated to the electron transport, carboxylation, and light-harvesting systems, which are closely related to the photosynthetic rate [34]. Relevant studies have shown that nitrogen deficiency decreases the capacity for electron transport, leading to a decline in the plant’s photosynthetic ability [35]. In this study, the addition of fulvic acid influenced the photosynthesis of rice leaves, exhibiting a certain positive effect on photosynthesis. All parameters increased with the concentration of fulvic acid. Specifically, the addition of fulvic acid increased the Pn of J 654 by 22.32–59.05%, Gs by 19.71–62.03%, Ci by 8.32–17.93%, and Tr by 10.21–33.10%; Pn of J 100 by 23.99–52.77%, Gs by 16.84–45.19%, Ci by 8.43–20.13%, and Tr by 8.48–39.93%. Among these, the Pn, Gs, Ci, and Tr under T3 and T4 treatments consistently remained at higher levels.

Nitrogen assimilation plays a crucial role in the life activities of plants, directly affecting their growth and development. Rice roots utilize various transporters to absorb nitrate (NO_3_^−^), ammonium (NH_4_^+^), and other nutrients from the soil. Research on nitrogen nutrition in rice has primarily focused on NH_4_^+^. However, since a portion of the ammonia fertilizer applied in the fields undergoes nitrification at the rice roots, studies on nitrate nitrogen in rice are also of significant importance. The NO_3_^−^ absorbed by plants is assimilated with the assistance of NR, and the final product NH_4_^+^ is assimilated through GS [36]. Studies have shown that the application of humic substances to maize seedlings can promote nitrogen metabolism [37]. Furthermore, studies have shown that the combined application of urea and fulvic acid solution can significantly enhance the activity of nitrogen metabolism-related enzymes in maize [38]. In this study, the addition of fulvic acid increased the activities of NR and GS. Under the same number of days, the NR and GS activities of J 654 and J 100 in the T3, T4, and T5 treatment groups were significantly higher than those in the T1 treatment group, while the NA and GS activities in the T4 treatment group consistently remained at a high level. When the concentration of added fulvic acid is 90 mg L^−1^, it is conducive to increasing the nitrogen metabolism and promoting the absorption of NO_3_^−^ and the assimilation of NH_4_^+^. GOT and GPT are important transaminases in the nitrogen assimilation process in plant roots and leaves. Additionally, Glu can be converted into Asp and Ala through GOT and GPT, respectively. Asp is an additional NH_4_^+^ detoxification molecule and a precursor for the synthesis of branched-chain amino acids (BCAAs). BCAAs derived from the Asp pathway (such as Met, Thr, and Ile) provide precursors for many plant secondary metabolites [39]. In this study, the addition of fulvic acid increased the activities of GOT and GPT. Compared to the T1 treatment, J 654 and J 100 consistently showed greater increases in GOT and GPT activities in the T3 and T4 treatments. This further indicated that an appropriate concentration of fulvic acid is beneficial for maintaining nitrogen metabolism levels.

Plants have several mechanisms that allow them to alleviate stress under abiotic stress conditions. One of these mechanisms is the enhancement of antioxidant enzyme activities, such as SOD, POD, and CAT, which reflect the plant’s ability to scavenge ROS. CAT and POD can metabolize H_2_O_2_, while SOD can catalyze the disproportionation reaction of O_2_^−^ with H_2_O_2_ [40]. MDA can bind to proteins in the cell membrane structure, inactivating the related proteins, and its content can be used to reflect the degree of lipid peroxidation [41]. In this study, the addition of fulvic acid increased the activities of SOD, POD, and CAT and decreased the production rate of O_2_^−^, as well as the H_2_O_2_ and MDA content. This indicated that fulvic acid alleviated the stress on plants and enhanced the activity of antioxidant enzymes, thereby improving the capacity to scavenge ROS. Compared with other treatments, J 654 and J 100 exhibited greater SOD, POD, and CAT activities in T3 and T4 treatments, while the O_2_^−^ production rate, H_2_O_2_, and MDA contents were lower. This indicated that an appropriate concentration of fulvic acid can better alleviate the stress on plants and maintain the scavenging capacity of ROS.

Proline is an amino acid that has highly beneficial effects in plants, aiding in the stabilization of subcellular structures, scavenging free radicals, and buffering the cellular redox potential under various stress conditions [42]. Under stress conditions, proline exhibits antioxidant properties that protect cell membranes, and its increase helps to maintain plant growth. The accumulation of soluble sugars is crucial for mechanisms such as repairing and compensating for cell volume loss, reducing damage caused by free radicals to cells, and protecting and stabilizing enzymes and membrane structures [43]. In this study, the addition of fulvic acid decreased the content of proline and soluble sugars. Under T3 and T4 treatments, the quantity of the proline and soluble sugars content remained consistently low in J 654 and J 100. This indicated that fulvic acid plays a crucial role in inducing stress resistance. Free amino acids in plants are the fundamental units constituting proteins and also serve as precursors for functional compounds, being transported within the plant in the form of nitrogen assimilates. Soluble proteins participate in various metabolic activities, and their content can reflect changes in nitrogen metabolism [44]. Compared to the T1 treatment, J 654 and J 100 showed a significant increase in free amino acids and soluble protein content in the T3 and T4 treatments. This also indicates that the addition of fulvic acid enhances the assimilation of inorganic nitrogen and photosynthetic capacity, thereby laying a solid foundation for material production.

## 4. Materials and Methods

### 4.1. Test Materials

The tested rice was of the commonly promoted varieties Jikedao 654 (J 654) and Jiyang 100 (J 100) from Jilin City, Jilin Province. Both are rice varieties with similar growth periods, excellent traits, and strong disease resistance, classified as medium–late maturing rice varieties (maturing within approximately 150 days). Both varieties exhibit high nitrogen fertilizer agronomic-use efficiency across a gradient of 0–210 kg/ha of pure nitrogen fertilizer and are classified as nitrogen-sensitive varieties.

### 4.2. Experimental Design

Rice seeds were sterilized with a 30% (volume fraction) sodium hypochlorite solution for 30 min, followed by rinsing with distilled water. After germination in the dark at 25 °C for 3 days, the rice seedlings were transferred to a plant growth chamber with a 14 h light and 10 h dark cycle and a diurnal temperature fluctuation between 28 °C and 20 °C. The relative humidity was maintained at 70%. They were cultured in clear water for 7 days, then replaced with 1/2 concentration Kimura B nutrient solution for another 7 days, followed by a complete concentration Kimura B nutrient solution for 14 days. Among them, the nutrient solution is renewed every 3 days. After 28 days of rice growth, they were subjected to low-nitrogen (NO_3_^−^ 0.5 mM and NH_4_^+^ 0.5 mM) treatments with different concentrations of fulvic acid (yellow fulvic acid): 0 mg L^−1^ (T1), 30 mg L^−1^ (T2), 60 mg L^−1^ (T3), 90 mg L^−1^ (T4), 120 mg/L (T5), and 150 mg L^−1^ (T6). The entire process spanned 15 days, with sampling and indicator measurements conducted every 5 days during this period. Each treatment was conducted in triplicate, meaning that three independent hydroponic boxes were used, with each box cultivating 200 rice plants. In addition, pH buffer 2-morpholinoethanesulfonic acid (MES) was added to the nutrient solutions containing low-nitrogen treatment and different concentrations of fulvic acid (T2–T6). The fulvic acid used was purchased from Shanghai ACME Biochemical Co., Ltd., China. The chemical molecular formula of fulvic acid is C_14_H_12_O_8_, with a molecular weight of 308.24. The pH value of a 10% fulvic acid aqueous solution is approximately 2.5–3.0.

### 4.3. Measurement Indicators and Methods

Agronomic traits

After 15 days of treatment under different concentrations of fulvic acid, rice seedlings were sampled. We selected three rice seedlings with essentially identical growth conditions from each hydroponic box, totaling nine seedlings per treatment, to measure the plant height and root number. The plant samples were dried at 105 °C for 30 min, then dried at 80 °C for 3 days, and the total dry weight of the plants was measured.

Chlorophyll content

After 15 days of treatment under different concentrations of fulvic acid, rice seedlings were sampled. We selected three rice seedlings with essentially identical growth conditions from each hydroponic box, totaling nine seedlings per treatment, to measure chlorophyll content. The chlorophyll content in rice leaves was calculated using the 90% ethanol extraction method based on the absorbance at 649 nm and 665 nm [45]. The chlorophyll a (Cha), chlorophyll b (Chb), and total chlorophyll content (Chl) concentrations were calculated with the following formulas: Cha = 13.95 * A665–6.8 * A649; Chb = 24.96 * A649–7.32 * A665; and Chl = Cha + Chb.

Photosynthesis-related parameters

After 15 days of treatment under different concentrations of fulvic acid, three rice seedlings with basically the same growth conditions were selected from each hydroponic box, totaling nine seedlings per treatment. Measurements were conducted on the uppermost fully expanded leaves using a portable photosynthesis system (Li-6800, LI-COR, Lincoln, Northeast USA) between 8:00 am and 11:00 am. Its net photosynthetic rate (Pn), stomatal conductance (Gs), transpiration rate (Tr), and intercellular CO_2_ concentration (Ci) were measured, with the light intensity in the leaf chamber set at 1200 μmol·m^−2^s^−1^.

Activity of nitrogen metabolism-related enzymes

Rice leaves were sampled after 5, 10, and 15 days of treatment under different fulvic acid concentrations. We selected three rice seedlings with basically the same growth conditions from each hydroponic box, totaling nine seedlings per treatment. The activity of NR was determined using the method described by Hageman and Reed [46]. The activity of GS was determined using the method described by Zhang et al. [47]. The activities of GOT and GPT were determined according to Wu et al. [48].

Antioxidant enzyme activity and substance content

Rice leaf samples were taken after 5, 10, and 15 days of treatment under different concentrations of fulvic acid. We selected three rice seedlings with basically the same growth conditions from each hydroponic box, totaling nine seedlings per treatment. The activities of SOD, POD, and CAT were determined according to Li et al. [49]. The MDA content was measured using the thiobarbituric acid method [49]. The O_2_^−^ production rate and H_2_O_2_ content were measured according to the method by Zhang [50]. The proline content was determined based on the method by Li [51].

After 21 days of treatment under different concentrations of fulvic acid, we selected three rice seedlings with basically the same growth conditions from each hydroponic box, totaling nine seedlings per treatment. The total nitrogen content was determined using the micro-Kjeldahl method [50].

### 4.4. Statistical Analysis

Preliminary data organization and analysis were conducted using Excel 2023 software. Data analysis was performed using SPSS 23.0 software. The least significant difference (LSD) test was employed, with *p* < 0.05. Graphing was carried out using Origin 2020 software, and correlation analysis and plotting were conducted using the “gpairs” package in R language 4.4.3 software.

## 5. Conclusions

To clarify the effects of different concentrations of fulvic acid on rice seedlings under low-nitrogen stress, the impacts of various fulvic acid concentrations on their agronomic traits, photosynthetic characteristics, activities of nitrogen metabolism-related enzymes, and antioxidant properties were evaluated. The results indicated that a fulvic acid concentration of 60–90 mg L^−1^ can promote plant growth and development, increasing the plant height, root number, total dry weight, and nitrogen accumulation in rice. It also significantly increased the content of photosynthetic pigments and enhanced the ability to capture light energy, thereby improving Pn, Gs, Ci, and Tr. The addition of fulvic acid also enhanced the activities of key enzymes in the leaf nitrogen metabolism, including NR, GS, GOT, and GPT, thereby maintaining the capacity for nitrogen metabolism. It decreased the toxic effects of ROS on plants, increased the activities of SOD, POD, and CAT, and decreased the production rate of O_2_^−^, as well as the H_2_O_2_ and MDA content. In addition, fulvic acid also improved osmotic regulation by alleviating stress, thereby leading to a reduction in the accumulation of proline and soluble sugars. Among these, fulvic acid promoted an increase in the content of free amino acids and soluble proteins, providing a solid material foundation for the growth and development of rice. In summary, when a quantity of fulvic acid between 60 and 90 mg L^−1^ is added, it can alleviate the inhibitory effect of low nitrogen on rice growth and enhance rice’s ability to adapt to low-nitrogen conditions. Our research results can provide a theoretical basis for the application of fulvic acid under low-nitrogen stress and for rice growth and resource utilization. Considering factors such as soil type, organic matter content, and field water management, future field trials should verify whether the application of fulvic acid can achieve similar benefits in soil–rice cultivation systems under a decreased nitrogen fertilizer application.

## Data Availability

The original contributions presented in the study are included in the article; further inquiries can be directed to the corresponding authors.

## Supplementary Materials

The following supporting information can be downloaded at: https://www.mdpi.com/article/10.3390/plants14182892/s1, Table S1: Percentage changes in NR, GS, GOT, and GPT under other treatments compared to T1 treatment. Table S2: Percentage changes in SOD, POD, CAT, O_2_^−^, H_2_O_2_, and MDA under other treatments compared to T1 treatment. Table S3: Percentage changes in soluble sugars, soluble proteins, free amino acids, and proline under other treatments compared to T1 treatment.
